# Unconventional two-dimensional vibrations of a decorated carbon nanotube under electric field: linking actuation to advanced sensing ability

**DOI:** 10.1038/s41598-017-12647-2

**Published:** 2017-10-18

**Authors:** Belisa R. H. de Aquino, Mehdi Neek-Amal, Milorad V. Milošević

**Affiliations:** 1 Department of Physics, Universiteit Antwerpen, Groenenborgerlaan, 171, B-2020 Antwerpen, Belgium; 2 Department of Physics, Shahid Rajaee Teacher Training University, Lavizan, 16875-163 Tehran, Iran

## Abstract

We show that a carbon nanotube decorated with different types of charged metallic nanoparticles exhibits unusual two-dimensional vibrations when actuated by applied electric field. Such vibrations and diverse possible trajectories are not only fundamentally important but also have minimum two characteristic frequencies that can be directly linked back to the properties of the constituents in the considered nanoresonator. Namely, those frequencies and the maximal deflection during vibrations are very distinctively dependent on the geometry of the nanotube, the shape, element, mass and charge of the nanoparticle, and are vastly tunable by the applied electric field, revealing the unique sensing ability of devices made of molecular filaments and metallic nanoparticles.

## Introduction

Nanoelectromechanical systems (NEMS) exhibit profound effects stemming from the coupling of the mechanical properties of the devices to their electronic degrees of freedom^1–5^. NEMS have been intensively studied and have an incredible potential for technological applications, e.g., the detection of external electromagnetic fields, control and enhancement of the high quality factor of the resonators and tunable high natural mechanical frequencies^5–7^. The latter makes nanoresonators candidates for advanced mass and force detectors, since small quantities of adsorbed mass can be detected by distinct shifts in the frequency of vibrations^8–15^. In that sense, doubly-clamped carbon nanotubes (CNTs), as well as cantilevers, have attracted noticeable attention since they offer unique resonance properties that are proven useful to detect atoms and single molecules^16–18^, which can be applied, among other possibilities, in mass spectrometry and air pollution control^19–21^. Furthermore, such precise mass sensing enables other biotechnological applications, e.g., DNA sequencing and detection of biomarkers at early stages of cancer and other diseases^22,23^. The electromechanical properties of CNTs typically allow to reach frequencies in the GHz range. Higher frequencies, i.e., up to the order of THz, are expected when suitable coating materials are used^24–28^. Moreover, CNTs are now able to present high quality factors, which combined with high resonance frequencies are essential for applications in mass sensing^28–30^.

On the other hand, metallic nanoparticles (NPs) have found numerous applications in engineering, materials science, physics, chemistry, biology and medicine^31–35^. They have an incredible potential for application in diagnostic imaging and drug delivery systems. Metallic NPs can attach to different organic compounds and antibodies with high selectivity and specificity, which can be improved by a ligand conjugation. The development of materials with antibacterial properties based on metallic NPs and studies of interaction of metallic NPs with HIV-1 have been already reported^36,37^. This versatility of NPs, and their relatively easy functionalization open a possibility to synthesize CNTs decorated by metallic NPs, and thereby broaden and advance NEMS applications^38–40^.

Traditionally the resonance frequency of CNTs is obtained from the one-dimensional (1D) oscillator model, characterized by a single frequency, where several definitions have been used for the effective spring constant *κ* ^2–8^, each of which adequate for a specific setup. Such 1D description is not sufficient, since it is known that applied electric field as well as molecular adsorption may induce nonlinear effects that entirely change the nanoelectromechanical resonant properties^41,42^, calling for further advanced theoretical models.

Here we thoroughly analyse the vibrational properties and the induced deflections of a doubly clamped CNT decorated with a charged metallic NP and subjected to an external electric field. We reveal that such a system exhibits nontrivial and diverse 2D vibrational motions depending on the strength of the electric field, and we propose a suitable theoretical model incorporating two frequencies of vibrations, i.e. the conventional frequency and the modulation frequency. Significant shifts in those frequencies were found for CNTs with different chiralities and for NPs made of different elements, which can be further employed for engineering nanoparticle diagnosis for biomedical applications based on nanomechanical sensors.

## Results and Discussion

### Field-induced deformations

**Figure 1:**
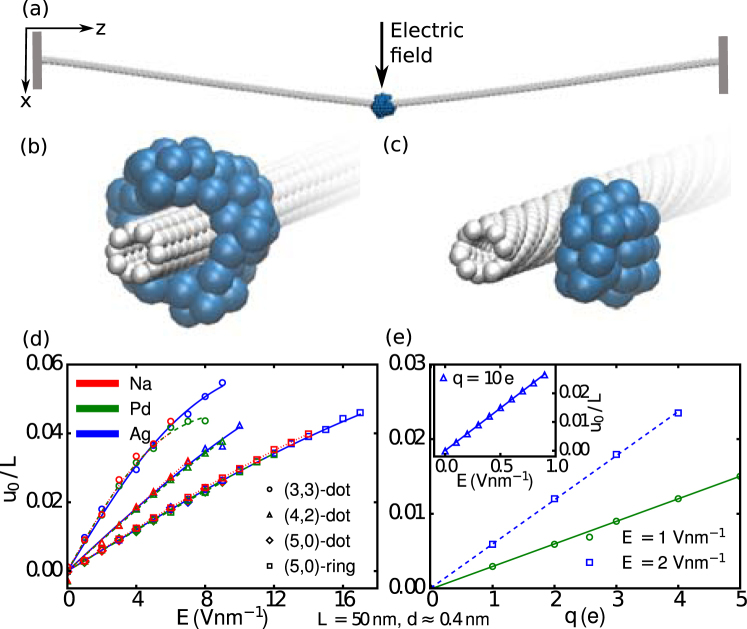
(**a**) The clamped CNT with a charged NP attached, bent under applied electric field. (**b**) A CNT decorated with a ring-shaped NP (100 atoms), and (**c**) with a dot-shaped NP (50 atoms) (zoomed in). (**d**) The variation of the maximal deflection of CNTs midpoint (*u*_0_) relative to the CNT length (*L*) with electric field (*E*), and (**e**) the NP charge *q*. The inset in (**e**) shows variations of *u*_0_/*L* with *E* for *q* = 10 e, while *q* = 1 e in (**d**). The lines plotted in (**d**,**e**) are the best fits obtained using Eq. (1).

Once a CNT decorated with a charged metallic NP is subjected to an external electric field (*E* = *E*_*x*_) applied perpendicularly to its axis (the *z* axis), it is bent [see Fig. 1a] and actuated into vibrations by the force acting on the charged NP. Our system can be modelled as a massive beam (of length *L*, aligned to the *z* axis, and fixed at both ends) subjected to a central force (*F*_*e*_ = *qE*) and stretched by a tension *T*. The maximal deflection at the midpoint of the beam, where the NP is located, is then given by ^3^:

1 $${u}_{0}(z=L\mathrm{/2)}=\frac{{F}_{e}L}{4YI{\xi }^{2}}\,[1-\frac{\tanh \,(\xi L\mathrm{/2})}{\xi L\mathrm{/2}}],$$

where *Y* is the Young’s modulus, *I* is the area moment of inertia, and $\xi =\sqrt{T/YI}$. If the bending energy is dominant and there is residual tension, one can write *T* = *c*_1_ + *c*_2_ *E* ^2^ ^3^.

By performing atomistic molecular dynamics (MD) simulations, our detailed analysis of the NP formation on a CNT revealed two typical types: i) a ring-shaped NP which is wrapped around the nanotube (with *N* = 100 atoms) [see Fig. 1b], and ii) a dot-shaped NP which is attached on one side of the outer surface of the nanotube (*N* = 50) [see Fig. 1c]. The variations of *u*_0_/*L* with *E* for CNTs with chiralities (3,3), (4,2) and (5,0), all of them with diameter *d* ≈ 0.4 nm and length *L* = 50 nm, decorated with a Na, Pd or Ag NP with charge *q* = 1 e are shown in Fig. 1d. Notice that the ring-shaped NPs remain attached to the CNT up to significantly higher electric field than the dot-shaped NPs. This is due to the larger number of atoms on the ring-shaped NP and the larger number of bonding sites between CNT and NP favored by the ring geometry. As the electric force actuating the CNT is proportional to the charge of the NP, NPs of different chemical elements but having equal charges induce nearly identical maximal deflections. However, for higher electric fields and consequently higher tension in the CNT, the different built-in tension on the CNT due to the different chemical elements of the NP can lead to slightly different deflections, as can be seen in Fig. 1d for the (3,3) CNT in the range of fields between 3 V nm^−1^ and 8 V nm^−1^. Although *u*_0_ is not very sensitive to the chemical element of the NP, it depends significantly on the CNT chirality, due to the different bending stiffness of such CNTs. The observed behaviour can be modelled by the continuum beam theory and captured by Eq. (1) – using two fitting parameters *a* = 4*c*_1_/*qL* and *b* = 4*c*_2_/*qL* – as shown by solid and dashed lines in Fig. 1d. For example, the fitting parameters for the (5,0) CNT in Fig. 1d were found to be *a* ~ 25 V nm^−2^ and *b* ~ 13 V^−1^.

For the (5,0) and (4,2) CNTs, *u*_0_ is linearly increasing with increasing the electric field whereas for the (3,3) CNT (armchair) the linear response is observed only for the small deflections and small electric fields. Furthermore, *u*_0_ can be tuned by varying the charge of the NPs, i.e. the larger the charge the larger the exerted force on the tube for the given field, hence larger the *u*_0_. In Fig. 1e the variation of *u*_0_/*L* with charge up to *q* = 5 e is depicted. The variations are shown for two (relatively large) values of electric field (*E* = 1 V nm^−1^ and *E* = 2 V nm^−1^) applied on a (5,0) CNT decorated with a dot-shaped Ag NP. For this particular case, the system responds linearly to the external force, i.e. *u*_0_ ∝ *qE*. The lines in Fig. 1e are two linear fits that yield the spring constant of the tube. We note that one can achieve similar and larger deflections of the CNT by using weaker electric field, by simply using a NP with larger charge. We verified this for a NP with *q* = 10 e on the (5,0) CNT and *E* < 1 V nm^−1^, as plotted in the inset of Fig. 1e. This goes on to prove the high degree of tunability of our hybrid device and its performance.

CNTs with larger diameter have smaller deflections, which is attributed to their larger bending stiffness. Compared to the results for the (3,3) CNT, we found a decrease in *u*_0_ of about 35% and 45% at *E* = 9 V nm^−1^ for (4,4) (*d* = 0.54 nm) and (5,5) (*d* = 0.68 nm) CNTs, respectively. The deformations of the CNTs also depend on the length of the tubes. For example, we found a linear increase of *u*_0_ with increasing the length of the (5,0) CNT decorated with a dot-shaped Ag NP (*N* = 50 atoms, *q* = 1 e) and two different applied electric fields, i.e. *E* = 1 V nm^−1^ and *E* = 2 V nm^−1^. The latter are in agreement with the predictions of Eq. (1) ^3^. Notice that such nanoscale deflections are measurable using atomic force microscopy^43^.

### Two dimensional vibrations

**Figure 2:**
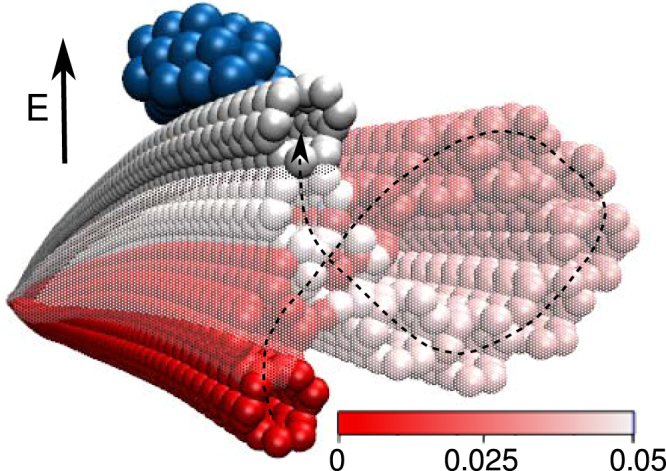
Cross-sectional view (55% of the CNT length) of the two-dimensional motion of the (3,3) CNT (*L* = 50 nm) decorated with a dot-shaped Ag NP (*N* = 50, *q* = 1 e), subjected to *E* = 4 V nm^−1^ (direction indicated by the arrow). The colour bar represents the simulation time in nanoseconds.

The presence of two spatially orthogonal vibration modes allows the CNT to vibrate along both directions even when subjected to a time-independent and constant electric field, which is due to a nonlinear interaction between the two vibration modes^41^. Moreover, this effect can be enhanced by the ever present asymmetry due to the attachment of a NP, which can also lead to changes in the orthogonal frequencies of vibration^42^. This is particularly relevant to our case, due to the not ideally symmetric positions of the metallic atoms within the NP (even the atoms of a ring-shaped NP do not form a perfect circular ring), as well as the asymmetry of the boundary conditions with respect to the chirality of the CNT on the clamped ends. Figure 2 depicts the two-dimensional motion of the (3,3) CNT decorated with a dot-shaped Ag NP subjected to *E* = 4 V nm^−1^.

**Figure 3:**
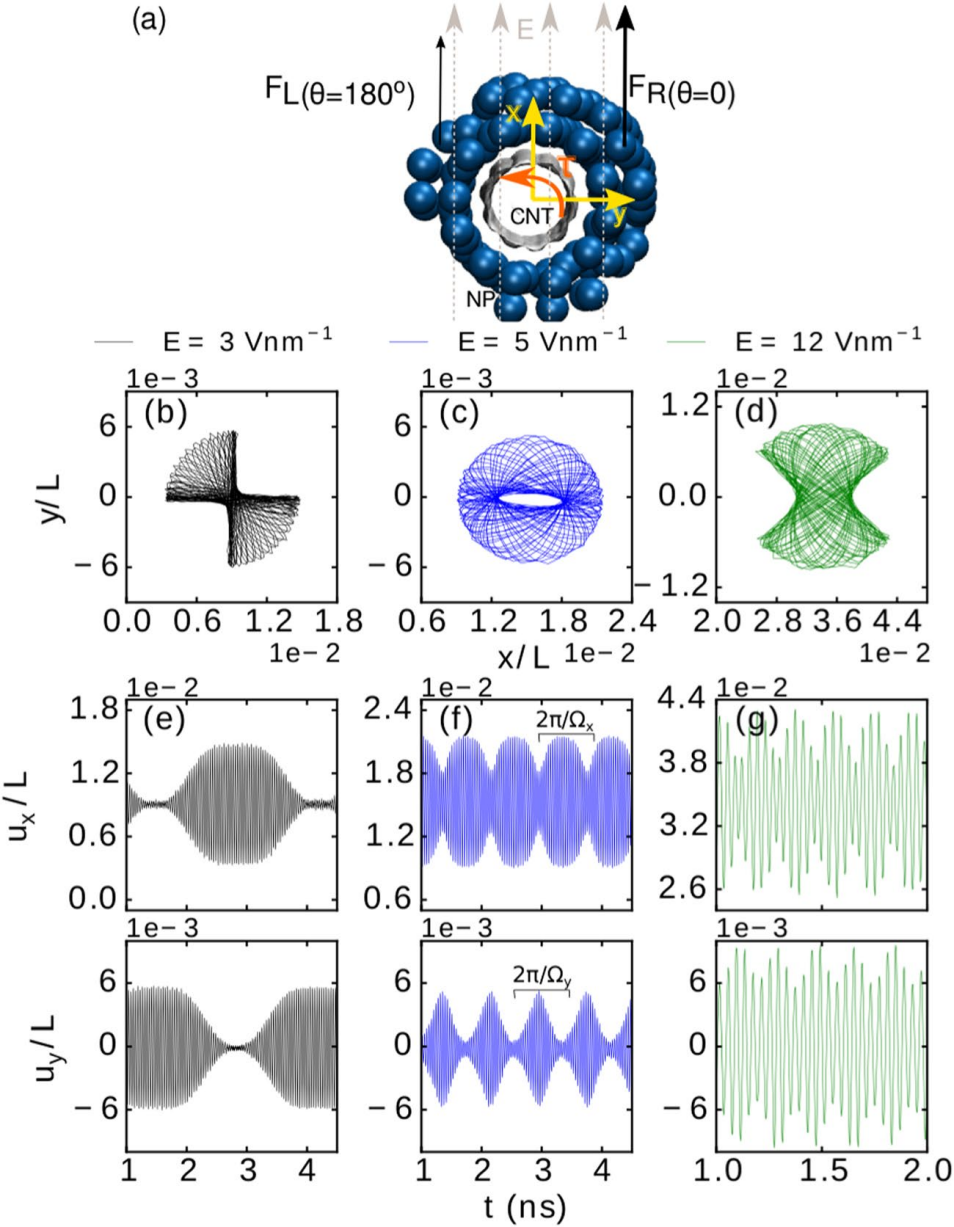
(**a**) Cross-sectional view of a decorated CNT, with indicated typical force components on the right and left side of the NP (*F*_*R*_ and *F*_*L*_ respectively), and the resulting torque *τ*. The path of vibrational motion in the (*x*, *y*) plane of the midpoint of a (5,0) CNT with *L* = 50 nm decorated with a ring-shaped Ag NP of 100 atoms and *q* = 1 e, subjected to electric field *E* = 3 V nm^−1^ (**b**), 5 V nm^−1^ (**c**), and 12 V nm^−1^ (**d**). The corresponding relative deflections *u*_*x*_/*L* and *u*_*y*_/*L* as a function of time are shown in panels (e–g), respectively. The envelope frequencies Ω_*x*_ and Ω_*y*_ are indicated in panel (f).

More generally by neglecting longitudinal vibrations, the deformation of the tube can be written as $\overrightarrow{u}={u}_{x}\hat{i}+{u}_{y}\hat{j}$, where

2 $${u}_{i}(\overrightarrow{r},E,t)={u}_{\mathrm{0,}i}(\overrightarrow{r},E)+{w}_{i}(\overrightarrow{r},E,{{\rm{\Omega }}}_{i},t)\,\cos \,({\omega }_{i},t),$$

with $\overrightarrow{r}$ being the position vector on the tube surface and *i* = (*x*, *y*). Here *w*_*i*_ is a periodic function (with angular frequency Ω_*i*_) indicating the envelope of the vibrational profiles in the *i* direction (see below), and *u*_0,*i*_ is the maximal deflection of the tube in the *i* direction. The *ω*_*i*_ is the angular frequency of vibrations along the *i* axis. Notice that for any given electric field as well as CNT and NP properties, we always found *u*_0,*y*_ = 0 and *u*_0,*x*_ = *u*_0_ in Eq. (2) [see Eq. (1)]. The non-uniform distribution of atoms in a ring-shaped NP causes the net force exerted on it (due to the electric field) to be different in, e.g., *θ* = 0 and *θ* = 180° directions, where *θ* is the polar angle at the circular cross-section of the tube at *z* = *L*/2 [see Fig. 3a, where *F*_*L*_(*θ* = 180°) < *F*_*R*_(*θ* = 0°)]. The latter results in a net torque (*τ*) and eventually a complex entangled and combined linear and rotational motion in the x-y plane. For direct visualization of the 2D vibrations, we refer the reader to the Supplementary Videos S1 and S2.

The aforementioned asymmetry in the microscopic structure of NPs may increase when large electric field is applied. In fact the atoms within the NP quickly rearrange once the electric field is switched on, e.g., noticeable changes on the structure of the ring-shaped Ag NP (*N* = 100, *q* = 1 e) on the (5,0) CNT (*L* = 50 nm) were observed for *E* ≥ 4 V nm^−1^ within the initial 30 ps of the simulation. We found that the NPs preserve the new arrangement during the rest of the simulation, in all considered cases. Although such change in the microscopic structure of the NPs is usually neglected in similar considerations to ours^8^, there were some experimental observations of the latter effect under specific circumstances reported by Conley *et al*.^41^ and Gil-Santos *et al*.^42^. We show here that this effect is much more important than previously assumed.

In Fig. 3b–d we depict three typical trajectories of the observed 2D vibrations of the midpoint of the (5,0) CNT decorated with a ring-shaped Ag NP with *q* = 1 e. Completely different trajectories were found for different applied electric fields. For *E* = 3 V nm^−1^ (b) and *E* = 12 V nm^−1^ (d) we obtained a non-circular (butterfly wings) pattern of trajectories, while an toroidal-shape trajectory pattern was found for *E* = 5 V nm^−1^ [see panel (c)].

The corresponding variations with time of *u*_*x*_(*z* = *L*/2, *E*, *t*) and *u*_*y*_(*z* = *L*/2, *E*, *t*) relative to *L* are shown in Fig. 3e–g, revealing different envelope frequencies of vibrations, i.e., Ω_*x*,*y*_, for different applied electric fields, e.g., Ω_*x*_/2*π* = 0.36 GHz, Ω_*x*_/2*π* = 0.94 GHz and Ω_*x*_/2*π* = 4.22 GHz for *E* = 3 V nm^−1^, *E* = 5 V nm^−1^ and *E* = 12 V nm^−1^, respectively. The correlation function 〈*u*_*x*_(*t*), *u*_*y*_(*t*)〉 = *δ* can reveal the extent in which the observed 2D vibrations contain linear (*δ* → 1) or perpendicular polarization (*δ* → 0). In most of the cases, for weak electric fields [e.g. see Fig. 3b] the *x* and *y* motions were found to have predominantly linear polarization (being correlated/anti-correlated (i.e. *δ* > 0/*δ* < 0)), while for larger electric fields (e.g. Fig. 3c,d) the motions have predominantly perpendicular polarization, i.e. become nearly uncorrelated. The complete set of found trajectory patterns and the corresponding calculated *δ* are shown in Supplementary Fig. S1.

**Figure 4:**
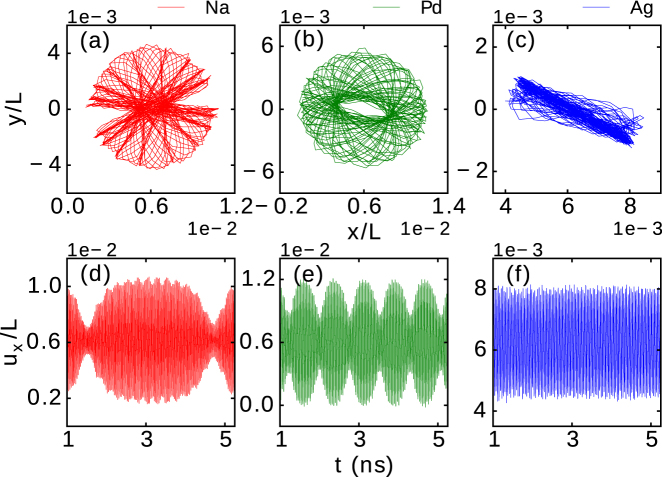
The path of vibrational motion in the (x, y) plane of the midpoint of a (5,0) CNT with *L* = 50 nm, subjected to the electric field *E* = 2 V nm^−1^ and decorated with a Na (**a**), Pd (**b**) and Ag (**c**) ring-shaped NP of 100 atoms and *q* = 1 *e*. The corresponding relative deflection *u*_*x*_/*L* as a function of time is shown in panels (d–f), respectively.

The trajectories of the observed 2D vibrations of the midpoint of the (5,0) CNT decorated with a ring-shaped NP with *q* = 1 e are shown in Fig. 4a–c for *E* = 2 V nm^−1^ and different constituent elements of the NP. For a Na NP (a) we obtained a circular fishnet trajectory pattern. For a Pd NP (b), a toroidal-shape trajectory pattern was found (similar to the pattern depicted in Fig. 3c for an Ag NP subjected to *E* = 5 V nm^−1^). In the case of an Ag NP subjected to *E* = 2 V nm^−1^ a linear pattern of trajectories was observed (c).

The envelope frequency primarily depends on the properties of the NP and its bonding to the CNT. We point out that NPs of different elements could not be discerned in our deflection analysis in Fig. 1d, but do lead to different 2D pattern of vibrations of the CNT in the last analysis. Therefore, different envelope frequencies are observed for different constituent elements of the NP, as shown in Fig. 4d–f, even when having nearly identical mass as in the case of Ag and Pd. The latter is mainly due to slightly different interactions between Pd and Ag atoms, which leads to a higher density of Pd NPs as compared to Ag NPs. Also, there is a stronger interaction between Pd NPs and the CNT, as the C-Pd equilibrium distance is smaller than that of C-Ag. The different induced local strain (where the NP is bonded to the CNT) causes a subtle yet distinctive difference in the elastic properties of the tube. Moreover, the two-dimensional CNT motion also relates to the properties of the nanotube, since different 2D trajectory patterns and envelope frequencies are also observed for CNTs of different chiralities.

The quality (Q) factor of the CNT-NP device can be determined by measuring the number of oscillations corresponding to the attenuation of the CNT vibrations. By increasing the electric field we found that the Q factor decreases, e.g., a (5,0) CNT decorated by a dot-shaped Ag NP (*N* = 50, *q* = 1 e) shows a decrease in the Q factor from *Q* = 3.5 × 10^4^ to *Q* = 7.6 × 10^3^. Furthermore, by increasing N we found decreasing Q, e.g., the Q factor of a (4,2) CNT decorated with a dot-shaped Ag NP decreases from *Q* = 6.5 × 10^4^ for *N* = 20 to *Q* = 7.0 × 10^3^ for *N* = 150. These results are in agreement with ref.^28^. In the case of CNTs decorated with ring-shaped NPs, we did not find significant attenuation in the CNT vibrations during our simulation time of *t* = 25 ns, confirming the possibility of achieving higher Q factors^29,30^.

**Figure 5:**
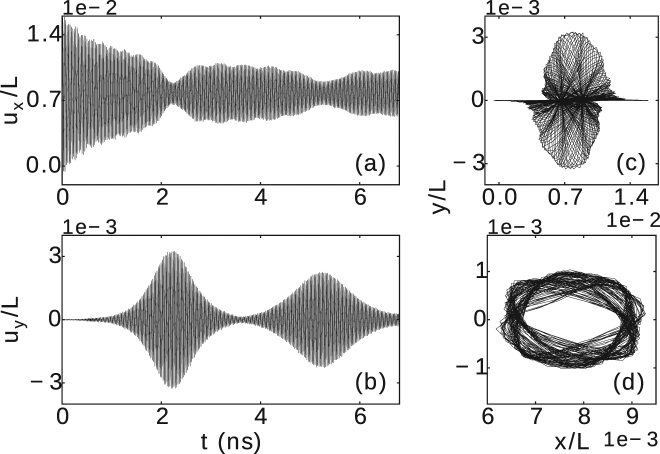
The variation with time of the relative deflections (**a**) *u*_*x*_/*L* and (**b**) *u*_*y*_/*L*, and the corresponding path of vibrational motion in the (x, y) plane of the midpoint of a pristine (5,0) CNT with *L* = 50 nm, *q* = 1 e, and subjected to the electric field *E* = 5 V nm^−1^, for a simulation time (**c**) under 3.5 ns and (**d**) in the period 17 ns–20 ns.

We emphasize that a pristine CNT has thermal vibrations at finite temperature. On the other hand, an external electric field induces charge on CNTs. By applying an electric field (in the absence of a NP), a corresponding electrostatic force is distributed along the CNT instead of a central localized force (when a charged particle is located at the midpoint of a neutral CNT). Therefore, the 2D vibrations and trajectory patterns of a pristine CNT are expected to be different than that of a decorated CNT. We depict the relative deflections *u*_*x*_/*L* and *u*_*y*_/*L* of a pristine (5,0) CNT as a function of time in Fig. 5a,b, where a net charge *q* = 1 e distributed over the CNT is subjected to *E* = 5 V nm^−1^. We found different time evolution in comparison to the deflections of a (5,0) CNT decorated by a ring-shaped Ag NP (*N* = 100, *q* = 1 e), shown in Fig. 3f. For a pristine (5,0) CNT, initially the deflections in the x-direction are four times larger than those in the y-direction. The trajectory pattern for a pristine CNT during a simulation time under 3.5 ns is shown in Fig. 5c. However, the deflections in x and y directions reach the same order of magnitude at a later time. The trajectory pattern for 17 ns − 20 ns (Fig. 5d) shows that the deflections for the pristine CNT can be even 3 times smaller than the deflections of the decorated CNT (see Fig. 3c). As seen in Figs 3c and 5c,d, the trajectory patterns for a pristine and a decorated CNT are clearly different. Furthermore, the pristine CNT yields *u*_0_/*L* ≈ 7.7 × 10^−3^, while for the decorated CNT *u*_0_/*L* ≈ 1.5 × 10^−2^. Such differences also impact the resonance frequency of vibrations, e.g., the pristine CNT has a resonance frequency of about 35 GHz for *E* = 5 V nm^−1^, while a frequency of 27 GHz was obtained for the decorated CNT.

### Resonance frequency

**Figure 6:**
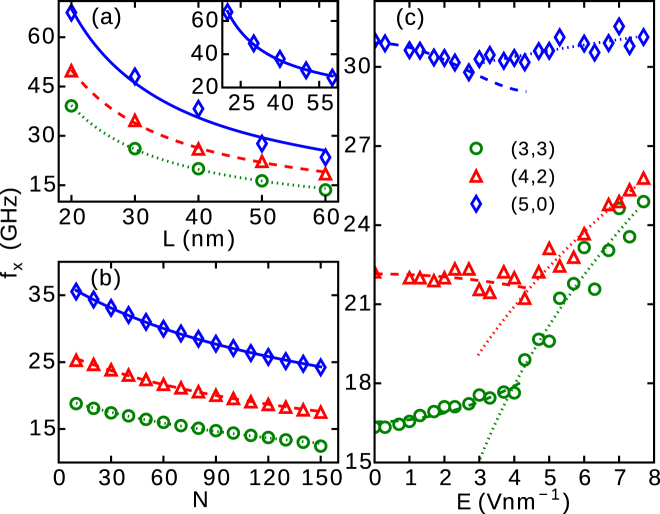
The variations of resonance frequency *f*_*x*_ with (**a**) length (*L*) of three different CNTs, (**b**) number of atoms in the NP (*N*) and (**c**) applied electric field (*E*). All CNTs were decorated with a dot-shaped Ag NP (*q* = 1 e). In (**a**,**b**) *E* = 0 while for the inset of panel (a) *E* = 2 V nm^−1^. In (**b**,**c**), *L* = 50 nm and in (**a**,**c**), *N* = 50. The lines plotted in (**a**,**b**) are fits using Eq. (3). The dashed and dotted lines in (**c**) are the fits obtained using empirical dependencies [see text].

Although we show there exist two frequencies characterizing the 2D vibrations, we found that the conventional frequencies (*ω*_*x*_) remain well described by traditional models. The frequency of vibrations along the *x* axis can be approximated as ^5,44^

3 $${f}_{x}=\frac{{\omega }_{x}}{2\pi }\approx \frac{1}{2\pi }\sqrt{\frac{192YI}{m{L}^{3}}}+\sqrt{\frac{T}{4mL}},$$

where *m* = 0.375*m*_0_ + *M* is the effective mass, with *m*_0_ and *M* being the mass of the CNT and the NP, respectively. Obviously, *f*_*x*_ should decrease with increasing *L*. The variation of *f*_*x*_ with *L* for three different CNTs is shown as data symbols in Fig. 6a for the case of a dot-shaped Ag NP with 50 atoms and *q* = 1 e and *E* = 0. We found that *f*_*x*_ still decreases with *L* when an electric field is applied, as shown in the inset of Fig. 6a for the (5,0) CNT subjected to *E* = 2 V nm^−1^. The plotted lines are fits according to the functional dependence ${c}_{3}\sqrt{{L}^{-3}}+{c}_{4}\sqrt{{L}^{-1}}$ (notice that *T* = *c*_1_ for *E* = 0).

The fit in Fig. 6b is made according to ${f}_{x}\propto 1/\sqrt{0.375m+\mu N}$, which explains very well the found behaviour of *f* with the number of atoms in the NP. Interestingly, the same fit holds for all three considered chairalities of the CNT with Ag NP, with a 10% spread on the value of *μ* ≈ (94–112). There are distinct shifts in the found frequency of vibrations for different CNTs, as shown in Fig. 6a,b, which are clearly enabling one to identify the chirality of the CNT. Furthermore, we also found frequency shifts when the atomic element or the overall mass of the NP was changed, for a given CNT. For instance, we found a frequency shift of about 1 GHz for two NPs with *M* ≈ 5.5 zg, but made of either 150 Na or 30 Ag atoms. For NPs with 150 atoms, a frequency shift of about 10 GHz was observed for the Na NP in comparison to Ag and Pd NPs (we note that the dependence of deflection *u*_0_(*E*) for given number of atoms in the NP did not show significant difference for different elements, see Fig. 1d). Finally, for NPs made of the same element, e.g. with 10 and 20 atoms of Ag (Δ*M* ≈ 2 zg), the frequency shift is found to be ~1 GHz. Such high sensitivity of our device is therefore rendered promising for mass-sensing applications, especially since the vibrational frequencies of CNTs above 1 GHz are readily experimentally detectable^25,28,45^. We note here that, since we neglected the polarization effects of the tubes, we expect similar results for non-metallic actin-filaments decorated with charged metallic NPs^46^, which yields a broader outlook of our analysis in the present paper.

The variations of *f*_*x*_ with *E* are more complicated. For three different CNTs, the MD data as a function of *E* is shown by symbols in Fig. 6c. By increasing *E*, one observes that transitions occur where *f*_*x*_(*E*) changes slope. We attribute this transition to the stretching becoming dominant over the bending regime^1^. The transition field *E*_*t*_ also varies with the CNT’s chirality, e.g. larger *E*_*t*_ was found for the (3,3) and (4,2) CNTs than for the (5,0) CNT. The transition can also shift with the number of atoms in the NPs, e.g. the (5,0) CNT decorated with a ring-shaped Ag NP (*N* = 100, *q* = 1 e) has a transition field *E*_*t*_ ≈ 6 V nm^−1^, while *E*_*t*_ ≈ 3 V nm^−1^ for the dot-shaped Ag NP (*N* = 50, *q* = 1 e).

Finally, we find that the behavior of *f*_*x*_(*E*) at low field is not the same for the (5,0), (4,2) and (3,3) CNTs, which is not captured by Eq. (3). To propose a functional dependence that can account for this behavior, one should consider various possible reasons for frequency attenuation (e.g. due to electric field counteracting the CNT’s restoring forces discussed in ref.^2^ as well as upward (higher-order) corrections for the frequency of each regime (see ref.^3^). We found that the best fits, after adding functional terms corresponding to the latter discussion, are obtained by empirical formulas $\sqrt{1+{c}_{5}{E}^{2}+{c}_{6}{E}^{4}}$ for the bending regime ($T\ll YI/{L}^{2}$) and $\sqrt{1+{c}_{7}{E}^{\mathrm{2/3}}}$ for the stretching regime ($T\gg YI/{L}^{2}$), shown respectively by dashed and dotted lines in Fig. 6c.

**Figure 7:**
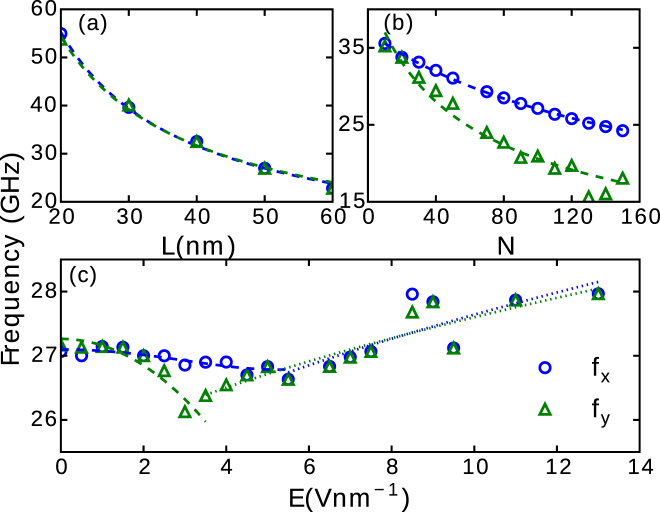
The variations of *f*_*x*_ and *f*_*y*_ with (**a**) *L*, for a ring-shaped NP and *E* = 2 V nm^−1^, (**b**) *N*, for a dot-shaped NP and *E* = 0, and (**c**) *E*, for a ring-shaped NP and *N* = 100. In all the panels a (5,0) CNT and a Ag NP (*q* = 1 e) are considered. The lines plotted in (**a**,**b**) are fits using Eq. (3). The dashed and dotted lines in (**c**) are the fits obtained using empirical dependencies [see text].

In addition to the frequency of vibrations along the direction of the electric field (*f*_*x*_) the entangled two-dimensional CNT vibrations present a transverse mode along the y direction with a frequency *f*_*y*_. In Fig. 7, the variations of the two frequencies *f*_*x*_ and *f*_*y*_ with *L*, *N* and *E* are shown for the (5,0) CNT. We found that the variation with length is nearly independent of the direction (*f*_*y*_ ≈ *f*_*x*_), as shown in Fig. 7a. However, lower *f*_*y*_ was observed when increasing *N* (see Fig. 7b). The variations of the two frequencies with E exhibit significant difference where the transition between the bending and the stretching regime occurs (see Fig. 7c), which indicates a more complex vibrational motion than those characteristic of either regime. Similar difference in the transverse frequency has been previously reported when varying the position of the NP on the CNT^42^. This suggests that symmetry breaking contributes to the attenuation of the transverse frequency, which justifies the difference between *f*_*x*_ and *f*_*y*_ when varying *N*.

In summary, we addressed high sensitivity of decorated nanoelectromechanical systems (NEMS) to the microscopic properties of the attached object. The deformations of such device made of a carbon nanotube (CNT) and a metallic nanoparticle (NP) under electric field were spatially mapped. The exhibited trajectory patterns for the two dimensional vibrational motions of the CNT were found to be unconventional and dependent on the strength of the applied electric field. Such 2D motions can not be described by the well known linear Euler-Bernoulli beam-mass theory, but exhibit also an envelope frequency (Ω) which was found to be an order of magnitude smaller than the conventional frequency, thus convenient for experimental detection and analysis. Both the conventional and the modulation frequency were shown to be distinctively sensitive on the CNT geometry and all properties of the NP (shape, element, mass and charge), enabling quite broad sensing applications of this device, and further development of biological/inorganic hybrid devices along similar ideas.

## Model and Methods

Our mechanical nanosensor, shown in Fig. 1a, comprises a charged metallic NP located around/at the outer midpoint of the doubly clamped carbon nanotube, and is subjected to an external constant electric field perpendicular to the nanotube. We investigate three types of CNTs of ≈0.4 nm diameter, i.e. (3,3) (armchair), (4,2) (chiral) and (5,0) (zigzag) CNTs. CNTs of small diameter constitute essentially ideal 1d systems and the existence of 0.4-nm CNTs has been theoretically predicted and experimentally confirmed^47,48^. In most of the here considered cases the length of the CNT was *L* = 50 nm, while the number of atoms within the NP and the charge of the NP were taken as *N* = 50–100 and *q* = 1–10 e, respectively. We considered three kinds of NPs, made of Na, Pd or Ag. To isolate the vibrations induced solely by the actuation due to the charged NP and external electric field, we neglected induced charges on the CNT^17^. In order to compare the deflections and vibrations of a decorated CNTs with those of a pristine CNT, we considered a charged (5,0) CNT with an uniform charge *q* = 1 e.

We employed atomistic MD simulations, with the Adaptive Intermolecular Reactive Empirical Bond Order (AIREBO) force field^49^ and the Embedded Atom Method potential (EAM)^50^ as implemented in the LAMMPS package^51^ to respectively model the carbon-carbon bonds and metal-metal interaction (for silver and palladium). The carbon-metal and the metal-metal interactions for sodium were modeled by the Lennard-Jones (LJ) potential with parameters taken from refs^52,53^.

CNT-NP composites can be synthesized either by chemisorption or physisorption, leading to different interfaces between the CNT and the NP. For example, the intensity of the adsorption energy and the nature of the adsorption site indicate that while Ag NPs are physisorbed, Pd NPs are chemisorbed, with stable covalent bonds due to a large contribution from d-orbitals^54–57^. However, the binding between the CNT and Ag or Pd NPs is relatively weak as compared to the metal-metal interaction^57,58^ enabling one to use the simple LJ potential in the model for the C-Ag and C-Pd interactions^58,59^. Please note that our method comprises classical simulations which allows us to simulate large systems and their behavior over long time.

## Electronic supplementary material

Video S1

Video S2

Supplementary Info
