# Supplementary material for: Unconventional two-dimensional vibrations of a decorated carbon nanotube under electric field: linking actuation to advanced sensing ability
Source: Sci Rep. 2017 Oct 18;7:13481. doi: 10.1038/s41598-017-12647-2 (PMC5647406; doi:10.1038/s41598-017-12647-2)
Supplement: Supplementary file 3 — Supplementary Info [file 41598_2017_12647_MOESM3_ESM.pdf]

Supplementary Information for
Unconventional two-dimensional vibrations of a decorated carbon nanotube under electric field: linking actuation to advanced sensing ability

Belisa R. H. de Aquino[1], Mehdi Neek-Amal[1], and Milorad V. Milošević[1]

[1]Departement Fysica, Universiteit Antwerpen, Groenenborgerlaan 171, B-2020 Antwerpen, Belgium

Videos S1 and S2 show the lateral and top views of the deflection and 2D vibrations of the (5,0) CNT decorated by a ring-shaped Ag NP and subjected to an electric field E = 16 Vnm-1.

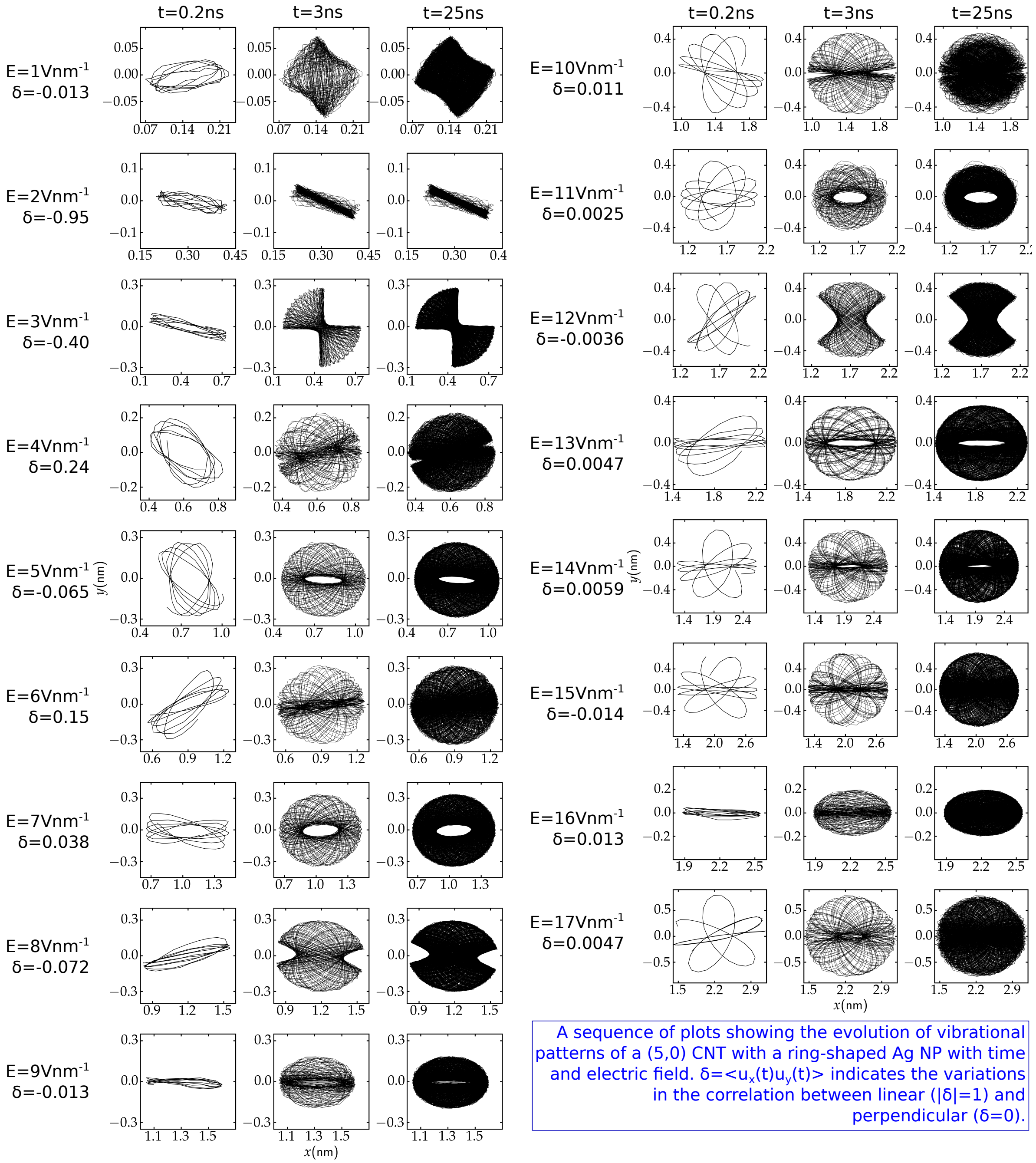

Figure S1: A sequence of plots showing the evolution of vibrational patterns of a (5,0) CNT with a ring-shaped Ag NP with time and electric field. $\delta =< u_x(t), uy(t) >$ indicates the variations in the correlation between linear ($|\delta| = 1$) and perpendicular ($\delta = 0$).
